# A retrospective comparative study of endoscopic and microscopic Tympanoplasty

**DOI:** 10.1186/s40463-018-0289-4

**Published:** 2018-07-04

**Authors:** Ying-Chieh Hsu, Chin-Lung Kuo, Tzu-Chin Huang

**Affiliations:** 1 Department of Otorhinolaryngology, Cathay General Hospital, 280 Ren-Ai Rd. Sec. 4, Taipei, Taiwan; 2 Department of Otolaryngology, Taoyuan Armed Forces General Hospital, Taoyuan, Taiwan, Republic of China; 3 Department of Otorhinolaryngology, Hsinchu Cathay General Hospital, Hsinchu, Taiwan

**Keywords:** Endoscopic ear surgery, Microscopic, Tympanoplasty, Cost-efficacy

## Abstract

**Background:**

This study compares endoscopic and microscopic tympanoplasty for the treatment of chronic otitis media (COM) without cholesteatoma.

**Methods:**

This retrospective study included 153 ears (139 patients) treated surgically (endoscopic or microscopic tympanoplasty) for COM in the absence of cholesteatoma at our hospital between January 2008 and October 2015. The adoption of transcanal endoscopic ear surgery (TEES) or microscopic ear surgery (MES) was divided temporally (before and since 2014). Comparisons between these groups focused on the following: (I) surgical outcomes, including successful tympanic membrane healing and post-operative complications; (II) restoration of hearing; and (III) consumption of medical resources, including the duration of surgery and anesthesia. All patients had a follow-up period of at least 3 months after surgery.

**Results:**

No statistically significant differences were observed between the two groups regarding surgical outcome or hearing restoration. TEES resulted in the successful healing of 96.2% of ear drums, whereas MES led to successful healing in 92% (*p* = 0.2826) of cases. The average hearing gains following surgery were 10.27 ± 6.4 and 12.43 ± 7.46 dB in TEES and MES, respectively. The consumption of medical resources in the TEES group was lower than that of the MES group (TEES versus MES) regarding the average operating time (87.8 ± 19.01 min (mins) versus 110.2 ± 17.0 (mins) (*p* <  0.0001)) and the mean duration of anesthesia ((for general anesthesia patients) (122.1 ± 21.25 mins versus 145.8 ± 16.88 mins) (*p* ≤  0.0001)).

**Conclusions:**

The results indicate that TEES can achieve surgical outcomes and hearing restoration comparable to those of MES. In addition, TEES appears to be associated with shorter surgical and anesthesia time, which makes it an ideal alternative for the management of COM without cholesteatoma.

**Trial registration:**

This study was approved by the Institutional Review Board of the Cathay General Hospital. (CGHIRB No: CGH-P105012).

## Background

A tympanoplasty is commonly performed to repair a perforated tympanic membrane and recover hearing loss in cases of chronic otitis media (COM) without cholesteatoma [1]. Conventional microscopic ear surgery (MES) using a post-auricular approach remains the most common tympanoplasty technique. However, it may require a large surgical incision, resulting in a visible scar and increased discomfort after surgery. Furthermore, the straight-line vision of microscopes greatly limits the surgeon’s ability to visualize the middle ear through the ear canal.

Endoscopic ear surgery (EES) was introduced in 1960, although it did not attract much attention initially [1]. However, the evolution of endoscopes and other instruments has made EES far more powerful, particularly in the management of ear disease [2]. Transcanal endoscopic ear surgery (TEES) permits wide-angle vision at a high resolution while enabling magnification of the structures of the middle ear as well as the direct visualization of hidden areas, such as the hypotympanum, sinus tympani, epitympanum, and posterior part of the mesotympanum [3–5].

The feasibility of using EES to manage diseases of the ear has been widely discussed. However, most prior studies have focused on the outcomes of EES in the management of cholesteatomas. Few studies have compared the efficacy of endoscopic versus microscopic tympanoplasty [2, 6, 7]. This paper reports our experience using tympanoplasties to treat COM without cholesteatoma and compares the surgical outcomes, hearing restoration rates, and medical resource consumption of MES and TEES.

## Methods

### Patients

This retrospective study included 153 ears of 139 patients who had COM without cholesteatoma and underwent tympanoplasty at Hsinchu Cathay General Hospital, Taiwan between January 2008 and October 2015. Patients who had perforated tympanic membranes, with or without concomitant tympanic cavity pathology such as granulation, fibrotic bands, shallow retraction pocket or ossicular chain defects, and underwent tympanoplasty were included in this study. Those who had cholesteatoma, cholesterol granuloma, or had tympanic membrane retraction pocket that underwent atticotomy or mastoidectomy were excluded. The patients were divided into two groups: conventional microscopic tympanoplasty group (MES group: 100 ears; 92 patients) and endoscopic tympanoplasty group (TEES group: 53 ears, 47 patients). The adoption of TEES or MES had a clear temporal division (before versus since 2014). In our hospital, all patients treated before 2014 received MES. As of January 2014, TEES was adopted as the primary procedure, and MES was used as a salvage technique for patients who were not suitable for TEES. The surgeon was prepared to switch to MES (through a post-auricular approach) in cases where the situation warranted, such as in the case of ear canal stenosis or uncontrollable hemorrhage during surgery that might interfere with transcanal endoscopic manipulation. The data were collected and analyzed from the hospital database.

All surgical procedures, post-operative follow-up evaluations and management were performed by the senior author (corresponding author) alone, which may avoid the bias related to surgeries and treatment performed by different surgeons. Furthermore, the review of medical records and data collection and analyses were performed by the first author and a research assistant who were blinded to the patients and their clinical management to reduce observer bias. This study was approved by the institutional board of the hospital.

### Exclusion criteria

Patients with a pre-operative or intra-operative diagnosis of cholesteatoma, cholesterol granuloma, and had tympanic membrane retraction pocket that underwent atticotomy or mastoidectomy were excluded. Moreover, patients who presented with facial paralysis or had a history of prior ear surgery were also excluded.

### Audiological assessment

All patients underwent pure tone audiometry (PTA) analysis to evaluate their pre- and post-operative hearing status. The mean hearing level and air-bone gap (ABG) of each patient were measured by averaging their hearing thresholds at 0.5, 1, 2, and 4 kHz.

### Anesthesia

Most of the tympanoplasties in our department were performed under general anesthesia (GA). Surgery was sometimes performed under local anesthesia (LA) for patients who were not suitable for GA for reasons such as old age, poor cardiopulmonary function or a difficult intubation. In some situations, LA was also performed in accordance with an individual patient’s preference or willingness.

### Surgical technique

All patients prior to 2014 underwent a conventional microscopic tympanoplasty for the treatment of COM without cholesteatoma, whereas all patients after January 2014 were managed with TEES. All surgical procedures, pre-operative assessments, and post-operative follow-up evaluations were performed by the senior author. In our department, TEES is indicated for almost all adult cases of COM without ear canal stenosis or congenital anomalies. These indications have been expanded to include the pediatric population, where TEES is initiated but the surgeon is prepared to switch to MES (post-auricular approach) if the situation warrants, such as in cases of ear canal stenosis or uncontrollable hemorrhage during surgery that might interfere with transcanal endoscopic manipulation.

In our hospital, all of the conventional microscopic tympanoplasties were performed via the post-auricular approach in order to obtain a wider surgical view. The procedure involves harvesting graft tissue from the areolar tissue layer above the temporalis fascia (loose areolar fascia) via a post-auricular incision. This is followed by the creation of a vascular strip in the ear canal, freshening the edges of the perforation, and the elevation of the tympanomeatal flap (TM flap) to gain access to the tympanic cavity. Following a thorough elimination of inflamed and infected tissue in the tympanic cavity, graft tissue is placed on the undersurface of the TM flap to reconstruct the tympanic membrane. Ossicular chains are assessed intra-operatively during all cases. If ossicular chain defects are noted during surgery, then a concomitant ossiculoplasty using an artificial total or partial ossicular replacement prosthesis (TORP or PORP) and cartilage may also be performed at the same time. Finally, the middle ear and external ear canals are packed with Gelfoam (absorbable gelatin sponge, USP, Pfizer, USA).

In contrast, TEES follows a transcanal approach using a rigid endoscope with an outer diameter of 3 mm and a length of 14 cm (HOPKINS II telescopes Karl Storz, Germany) at an angle of 0 or 30 degrees, in conjunction with a high definition (HD) video system. TEES at our facility involves the collection of graft tissue from three sources: areolar tissue above the temporalis fascia (through a post-auricular incision), cartilage from the perichondrium of the concha (through a retro-auricular wound), or the perichondrium of Tragus (from a wound within the ear canal). We then freshen the edges of the perforation, elevate the TM flap, and eliminate the inflamed and infected tissue in the tympanic cavity using an endoscope and various curved instruments, including needles, dissectors, and suction devices. Graft tissue is then placed on the undersurface of the TM flap to reconstruct the tympanic membrane. A concomitant ossiculoplasty with an artificial prosthesis (TORP or PORP) and cartilage may also be performed if an ossicular chain defect is noted during the surgery. The middle ear and external ear canals are also packed with Gelfoam at the end of the procedure.

### Post-operative follow-up

All patients returned for follow-up 1, 2, 4, and 8 weeks after surgery. External ear canal packing was removed within 2 weeks, and patients were followed-up every 2 weeks until the end of their recovery period. The integrity of the tympanic membrane was assessed and an audiogram with PTA was performed 3 months after surgery.

### Evaluation of outcomes

Retrospective medical record review and all subsequent data collection were conducted by an independent reviewer (the first author) who was not involved in the surgeries or post-operative follow-up, thereby diminishing possible observer bias. Analysis was performed by a research assistant who was blinded to the patients and interventions. The two groups were analyzed and compared from three perspectives: (I) surgical outcomes, including successful tympanic membrane healing and post-operative complications; (II) restoration of hearing function, including the average pre- and post-operative ABG, average hearing gain (dB), and percentage of patients with improved hearing (%, percentage of patients with post-operative hearing gain > 5 dB); and (III) consumption of medical resources, including the average time spent in surgery (mins) and the average time spent under anesthesia (mins).

### Statistical analysis

Statistical analysis was performed using SPSS for Windows (version 16.0; SPSS Inc., Chicago, IL, USA). Categorical variables were analyzed using a chi-squared or Fisher’s exact test. Differences between groups were analyzed using a Mann-Whitney U test. A *p*-value < 0.05 was considered statistically significant.

## Results

Our series included 139 patients (153 ears) (51 male (36.6%) and 88 females (63.3%)) between the ages of 6 and 78 years with a mean age ± standard deviation (SD) of 46.8 ± 14.5 years. Each subgroup presented with similar clinical and demographic characteristics (Table 1).

**Table 1:** Baseline clinical and demographic parameters

	TEES Group (*n* = 53 ears)	MES Group (*n* = 100 ears)	*p*-value
Sex			0.221
Male	22 (41.5%)	34 (34.0%)	
Female	31 (58.5%)	66 (66.0%)	
Lesion side			0.314
Left ear	30 (56.6%)	49 (49.0%)	
Right ear	23 (43.4%)	51 (51.0%)	
Average age ± SD (years)	48.9 ± 14.7	45.7 ± 14.2	0.1946
Average perforation size of tympanic membrane ± SD (%)^a^	51.6 ± 23.5	49.4 ± 21.1	0.5665
Comorbidity	7 (13.2%)	7 (7.0%)	0.2051
DM	4 (7.5%)	2 (2.0%)	
Hypertension	5 (9.4%)	4 (4.0%)	
Asthma	0	1 (1.0%)	
Hepatitis B	0	1 (1.0%)	
Average follow-up period ± SD (months)	3.5 ± 2.6	7.0 ± 10.4	0.016

^a^Perforation size was estimated through visual inspection of the percentage of perforation relative to the tympanic membrane area by the senior otologist (corresponding author)

### Comparison of surgical outcomes

Surgical procedures were adopted in accordance with the severity and extent of the pathology. The type of tympanoplasty was recorded and analyzed in accordance with a Wullstein classification of type I to V (Table 2) [8]. Since January 2014, TEES was adopted as the primary procedure for treating COM, and MES was used as the salvage technique for patients who were not suitable for TEES. The surgeons may need to switch to MES in cases where the situation warranted. However, none of the patients need to change to MES during the study period. It is also worth mentioning that 13 of MES patients required bony canalplasty because of insufficient surgical views whereas none of the patients in the TEES group required the procedure.

**Table 2:** Surgical procedure

	Group		p
	TEES (*n* = 53)	MES (*n* = 100)	
Graft material			< 0.0001
Areolar temporalis fascia	12 (22.6%)	100 (100%)	
Concha perichondrium	23 (43.4%)		
Tragus perichondrium	18 (34.0%)		
Tympanoplasty type			0.1094
Type I	46 (88.5%)	94 (94.0%)	
Type III	3 (5.8%)	5 (5.0%)	
Type IV	2 (3.8%)	1 (1.0%)	
Type V	1 (1.9%)	0	
Ossiculoplasty	6 (11.3%)	6 (6.0%)	0.127
PORP	3 (5.8%)	5 (5.0%)	
TORP	2 (3.8%)	1 (1.0%)	
Piston wire	1 (1.9%)	0	

The surgical outcomes of the two groups were compared in many categories. The rates of complete tympanic membrane healing in the TEES and MES groups were 96.2 and 92%, respectively (*p* = 0.282). We also compared the incidence of post-operative complications. The MES group included one patient with severe sensory neural hearing loss (SNHL) (SNHL > 70 dB), one patient with mastoiditis, and another patient with persistent otorrhea for 2 months after surgery (Table 3). We hypothesized that these three complications occurred due to a severe middle ear infection but were not relevant to the procedure itself. Overall, we were unable to detect any statistically significant differences (*p* <  0.05) between the two groups with respect to the rates of complete tympanic membrane healing or the incidence of post-operative complications.

**Table 3:** Surgical outcome

	Group		p
	TEES (*n* = 53)	MES (*n* = 100)	
Successful tympanic membrane healing	51 (96.2%)	92 (92.0%)	0.2826
Post-operative complications			0.2028
Severe SNHL	0	1 (1.0%)	
Mastoiditis	0	1 (1.0%)	
Persistent otorrhea (wet ear)	0	1 (1.0%)	

### Comparison of hearing outcomes

We also assessed the restoration of hearing in the two groups. The average hearing gain was 10.3 ± 6.4 dB in the TEES group and 12.4 ± 7.5 dB in the MES group (*p* = 0.1663). We did not detect any statistically significant differences (*p* <  0.05) between the two groups with respect to the average pre- and post-operative ABG, average hearing gain, or percentage of patients with improved hearing (Table 4).

**Table 4:** Restoration of hearing function

	Group		p
	TEES (*n* = 53)	MES (*n* = 100)	
Average pre-operative ABG ± SD (dB)	24.7 ± 8.1	25.0 ± 8.2	0.8217
Average post-operative ABG ± SD (dB)	14.4 ± 9.2	12.3 ± 6.2	0.2256
Average hearing gain ± SD (dB)	10.3 ± 6.4	12.4 ± 7.5	0.1663
Percentage of patients with improved hearing	51 / 53 (96.2%)	94 / 100 (94.0%)	0.4848

### Comparison of medical resource consumption

Statistically significant differences were observed between the two groups regarding the consumption of medical resources. Compared to the MES group, a higher percentage of patients in the TEES group was treated under LA (TEES versus MES: 17.0% versus 2.0%, *p* <  0.001), and a higher percentage of TEES was performed as outpatient surgery (TEES versus MES: 22.6% versus 1.0%, *p* <  0.0001). The mean duration of surgery was significantly shorter in the TEES group (87.8 ± 19.01 mins) than in the MES group (110.2 ± 17.0 mins, *p* <  0.0001), as was the mean duration of anesthesia (for GA patients) (mins) (122.1 ± 21.3 versus 145.8 ± 16.9, *p* ≤  0.0001) (Table 5). Overall, patients in the TEES group seemed to use fewer medical resources than those in the MES group with respect to shorter time spent in surgery and under anesthesia.

**Table 5:** Consumption of medical resources

	Group		p
	TEES (*n* = 53)	MES (*n* = 100)	
Outpatient/Admission			< 0.0001
Outpatient procedure	12 (22.6%)	1 (1.0%)	
Admission procedure	41 (77.4%)	99 (99.0%)	
Anesthesia method			0.0007
General (GA)	44 (83.0%)	98 (98.0%)	
Local (LA)	9 (17.0%)	2 (2.0%)	
Average surgical time ± SD (mins)	87.8 ± 19.0	110.2 ± 17.0	< 0.0001
Average time under anesthesia ±SD (mins) (GA patients)	122.1 ± 21.3	145.8 ± 16.9	< 0.0001

## Discussion

The main goals of a tympanoplasty for COM are to eradicate infection, repair the perforated tympanic membrane, and improve hearing [9]. For decades, MES was the main modality for ear surgery, enabling two-handed manipulation as well as binocular vision along with an excellent stereoscopic surgical view. However, the vision of a microscope may be limited when using a transcanal approach, particularly in hidden areas such as the anterior margin of the tympanic membrane and the sinus tympani or facial recesses, which forces the surgeons to use the post-auricular approach in order to obtain a wider surgical view. In some cases, creating sufficient space necessitates a canalplasty and soft-tissue retraction [5].

TEES provides an excellent surgical view, uses a smaller surgical incision, and preserves more tissue. Kozin et al. reported that a clear benefit existed for observational EES [2]. Some studies have suggested that when treating COM with cholesteatoma, TEES enables surgeons to avoid unnecessary mastoidectomies and prevent external ear canal widening and soft-tissue injuries during ear surgery [5–7, 10, 11]. Nonetheless, TEES still has a number of disadvantages, such as the need for one-handed manipulation, reduced endoscopic vision in the setting of uncontrollable hemorrhage, and the potential for thermal injury to the middle or inner ear caused by the endoscopic light source [12, 13]. However, operative EES is still in its infancy. Previous studies on EES have focused mainly on the management of cholesteatomas. Few researchers have conducted a systematic comparison of microscopic and endoscopic tympanoplasties in the absence of cholesteatoma [14–17].

### Surgical outcomes

In this study, the rate of successful tympanic membrane healing and the incidence of post-operative complications were similar in the TEES and MES groups. In the MES group, one patient presented with post-operative complication of severe SNHL, one had mastoiditis and another had persistent otorrhea for months after surgery. Overall, we observed no statistically significant differences in the surgical outcomes following MES and TEES. Choi et al. [14] concluded that the outcomes of microscopic and endoscopic tympanoplasties are similar, with graft success rates of 100 and 95.8% in the endoscopic and microscopic groups, respectively (*p* = 0.304). Dundar et al. [15] also reported that no significant differences in the condition of the graft were observed 12 months after surgery in pediatric patients who underwent a type 1 tympanoplasty through an endoscopic or microscopic approach (graft success rate: 87.5% (endoscopic) versus 94.3% (microscopic), *p* > 0.05). These outcomes are also consistent with those obtained in our study (graft success rate: TEES: 96.2% versus MES: 92.0%, *p* = 0.2826).

### Restoration of hearing function

Post-operative hearing restoration is another important indicator by which the outcomes of tympanoplasties can be evaluated. Dundar et al. [15] reported that there was no statistically significant difference in the pre-operative (20.40 versus 21.34 dB, *p* ≥ 0.05) and post-operative ABG (8.12 versus 8.13 dB, p ≥ 0.05) regardless of which procedure was performed. Similar to our results, we also observed no statistically significant differences between the TEES and MES groups in hearing restoration, including pre- and post-operative ABG, average hearing gain (dB) (10.3 ± 6.4 for TEES and 12.4 ± 7.5 for MES, *p* = 0.1663) and the percentage of patients with improved hearing after surgery (TEES versus MES: 96.2% versus 94.0%, *p* = 0.4848) (Table 3). This implies that TEES shows similar results to MES for the restoration of hearing after surgery.

### Consumption of medical resources

A medical record review found that most of the patients in the MES group were treated under GA. In contrast, a much higher percentage of cases in the TEES group utilized LA. In addition, a higher percentage of outpatient surgery was performed in the TEES group when comparing with the MES group. Our findings show that TEES can reduce the consumption of medical resources by shortening the surgical time, and the anesthesia time. The mean operative time in the TEES group (87.8 ± 19.01 mins) was significantly lower than that of the MES group (110.2 ± 17.0 mins). This result is consistent with previous studies that have reported that the operative time of an endoscopic tympanoplasty is significant shorter than that of a microscopic tympanoplasty [14, 16]. These differences may be attributed to differences in the surgical approach for TEES and MES. Prior to 2014, we employed a post-auricular approach in MES that produced a wide surgical wound during the surgery and required considerable time to manage the soft tissues and close the wound. In contrast, TEES is based on a transcanal approach, which leaves only a tiny wound associated with graft harvesting. The minimally invasive transcanal approach of TEES saves time in the assessment of the middle ear and results in far less soft-tissue damage, thereby reducing the time required to complete the surgery and the time spent under anesthesia. In addition, when performing microscopic tympanoplasty, a bony canalplasty may be required in narrow or crooked ear canal patients in order to obtain a sufficient surgical view. While in TEES, the endoscope can bypass the narrow part of the ear canal, provide greater visual access and wide-angle view of the middle ear, as well as sufficient manipulation space without the necessity of canalplasty, which can also help to save the surgical and anesthesia time significantly [5, 18].

TEES has also been shown to preserve the integrity of the outer ear and cause a less extensive injury to the cartilaginous ear canal, thereby reducing the incidence of post-operative complications such as tissue swelling, wound pain, bleeding, scaring, and ear canal stenosis [5, 18]. Choi also reported that patients who underwent an endoscopic tympanoplasty experienced far less pain the first day after the procedure than patients who underwent the microscopic procedure [14]. We infer that the minimally invasive nature of TEES can help to reduce the physical and psychological burden placed on patients, which may somewhat influence the patient’s reliance on hospitalization and explain why physicians opt to perform TEES under LA or as an outpatient procedure. Although some confounding factors exist, we believe that the results of our study reflect clinical reality to some extent.

According to previous studies, the success rate of microscopic tympanoplasties ranges from 90 to 98% [19, 20]. However, the outcomes of TEES still lack sufficient acceptance. In our series, we achieved a graft success rate of 96% following an endoscopic tympanoplasty, with satisfying improvements in hearing and no post-operative complications. Shoeb et al. [17] reported graft success rates of 93% using TEES and MES. Dundar et al. [15] also reported that in pediatric patients undergoing a type 1 tympanoplasty, the endoscopic and microscopic approaches appear to be equal in terms of hearing gain and graft success rate, whereas the operative duration was shorter in the endoscopic group than in the microscopic group. These findings are also consistent with those observed in this study. We therefore believe that TEES not only achieves surgical outcomes that are at least as good as those of MES but also reduces the consumption of medical resources due to a shorter procedure time. TEES is likely a good alternative to MES in the management of COM without cholesteatoma.

### Limitations

The selection bias in this study was minimized by adopting TEES and MES in a consecutive series, with a clear temporal division (before and since 2014) rather than one based on disease severity. When TEES was first adopted in our department in 2014, it was inevitable that part of the patients in the TEES group were within the learning curve of the operator which might cause bias in the results.

Furthermore, all surgical procedures, pre-operative assessments, and post-operative follow-up evaluations were performed by the senior author (corresponding author) alone, which avoids a discrepancy between different surgeons. However, this might increase the risk of bias in the review process.

In addition, this study was limited by a number of factors. This is a retrospective study conducted at a single hospital with relatively small group of patients. The sample size might be too small to come up with a widely accepted conclusion. A more extensive survey of cases or a multi-hospital study would be beneficial. The relatively short follow-up period in both groups likely led to an underestimation of actual long-term results. Although close office-based examination was performed after surgery, regular follow-up over a longer time scale should still be considered if necessary. Furthermore, several limitations remain regarding the use of TEES in the management of COM including that (1) stenosis, narrowing or exostosis of the external ear canal and (2) coagulopathies are relative contraindications of TEES due to the increased complexity of the endoscopic transcanal approach.

Two additional issues need to be addressed. First, we did not report the location of tympanic membrane perforation because the data were either incomplete or non-objective. Second, MES via endaural approach was not mentioned throughout the study because all of the microscopic tympanoplasties in our hospital were performed via the post-auricular approach.

## Conclusions

The results show that TEES can achieve satisfactory outcomes that are at least as good as those following traditional MES in the management of COM without cholesteatoma. Additionally, TEES appears to be associated with less consumption of medical resources in terms of shorter surgical and anesthesia time. However, further prospective studies should be conducted in the future to reinforce these conclusions.

## Abbreviations

ABG: Air-bone gap
COM: Chronic otitis media
MES: Microscopic ear surgery
PORP: Partial ossicular replacement prosthesis
PTA: Pure tone audiometry
TEES: Transcanal endoscopic ear surgery
TORP: Total ossicular replacement prosthesis

## References

[1] Zöllner F (1955). The principles of plastic surgery of the sound-conducting apparatus. J Laryngol Otol.

[2] Kozin ED, Gulati S, Kaplan AB, Lehmann AE, Remenschneider AK, Landegger LD (2015). Systematic review of outcomes following observational and operative endoscopic middle ear surgery. Laryngoscope.

[3] Thomassin JM, Duchon-Doris JM, Emram B, Rud C, Conciatori J, Vilcoq P (1990). Endoscopic ear surgery. Initial evaluation. Ann Otolaryngol Chir Cervicofac.

[4] Marchioni D, Alicandri-Ciufelli M, Piccinini A, Genovese E, Presutti L (2010). Inferior retrotympanum revisited: an endoscopic anatomic study. Laryngoscope.

[5] Tarabichi M (1999). Endoscopic middle ear surgery. Ann Otol Rhinol Laryngol.

[6] Presutti L, Gioacchini FM, Alicandri-Ciufelli M, Villari D, Marchioni D (2014). Results of endoscopic middle ear surgery for cholesteatoma treatment: a systematic review. Acta Otorhinolaryngol Ital.

[7] Marchioni D, Mattioli F, Alicandri-Ciufelli M, Presutti L (2009). Endoscopic approach to tensor fold in patients with attic cholesteatoma. Acta Otolaryngol.

[8] Wullstein H (1956). The restoration of the function of the middle ear, in chronic otitis media. Ann Otol Rhinol Laryngol.

[9] Sheehy JL, Anderson RG (1980). Myringoplasty. A review of 472 cases. Ann Otol Rhinol Laryngol.

[10] Marchioni D, Villari D, Alicandri-Ciufelli M, Piccinini A, Presutti L (2011). Endoscopic open technique in patients with middle ear cholesteatoma. Eur Arch Otorhinolaryngol.

[11] Ayache S, Tramier B, Strunski V (2008). Otoendoscopy in cholesteatoma surgery of the middle ear. Otol Neurotol.

[12] Bottrill I, Perrault DF, Poe D (1996). *In vitro* and *in vivo* determination of the thermal effect of middle ear endoscopy. Laryngoscope.

[13] Kozin ED, Lehmann A, Carter M, Hight E, Cohen M, Nakajima HH (2014). Thermal effects of endoscopy in a human temporal bone model: implications for endoscopic ear surgery. Laryngoscope.

[14] Choi N, Noh Y, Park W, Lee JJ, Yook S, Choi JE (2016). Comparison of endoscopic tympanoplasty to microscopic tympanoplasty. Clin Exp Otorhinolaryngol.

[15] Dündar R, Kulduk E, Soy FK, Aslan M, Hanci D, Muluk NB (2014). Endoscopic versus microscopic approach to type 1 tympanoplasty in children. Int J Pediatr Otorhinolaryngol.

[16] Lakpathi G, Reddy LS, Anand (2016). Comparative study of endoscope assisted myringoplasty and microscopic myringoplasty. Indian J Otolaryngol Head Neck Surg.

[17] Shoeb M, Gite V, Bhargava S, Mhashal S (2016). Comparison of surgical outcomes of tympanoplasty assisted by conventional microscopic method and endoscopic method. Int J Otorhinolaryngology Head Neck Surg.

[18] Tarabichi M, Presutti L, Marchioni D (2015). Principle of endoscopic ear surgery. Endoscopic ear surgery: principles, indications and techniques.

[19] Dornhoffer JL (1997). Hearing results with cartilage tympanoplasty. Laryngoscope.

[20] Indorewala S, Adedeji TO, Indorewala A, Nemade G (2015). Tympanoplasty outcomes: a review of 789 cases. Iran J Otorhinolaryngol.

